# Associations between social capital and screening mammography among older U.S. women

**DOI:** 10.1016/j.pmedr.2025.103026

**Published:** 2025-03-04

**Authors:** Tracy Huang, Christine C. Ekenga

**Affiliations:** Gangarosa Department of Environmental Health, Rollins School of Public Health, Emory University, Atlanta, GA, USA

**Keywords:** Social capital, Cancer, Screening mammography, Older adults, Mammograms

## Abstract

**Objective:**

To investigate the association between county-level social capital and screening mammography rates among older women in the United States.

**Methods:**

This cross-sectional ecological study included 2765 U.S. counties, using 2018 county-level screening mammography rates among female Medicare enrollees aged 67–69 as the outcome. Social capital data were obtained from the 2018 Social Capital Project, including indices for Family Unity, Institutional Health, Collective Efficacy, and Community Health. Multivariable log-binomial regression analyses were conducted to estimate adjusted prevalence ratios (aPRs) and confidence intervals for “high” mammography rates (top 10 % nationally), controlling for county-level demographic and healthcare covariates. Stratified analyses examined associations among metropolitan and nonmetropolitan counties.

**Results:**

Mammography screening rates ranged from 17 % to 64 %, with a mean of 41 %. Strong positive associations were observed between social capital and mammography rates (Q4 vs. Q1: aPR = 2.29, 95 % CI: 1.20–4.36), particularly for the dimensions of Community Health (Q4 vs. Q1: aPR = 1.99, 95 % CI = 1.25–3.17) and Institutional Health (Q4 vs. Q1: aPR = 4.31, 95 % CI = 2.40–7.75). These associations were strongest among nonmetropolitan counties. No significant associations were found for Family Unity or Collective Efficacy.

**Conclusions:**

County-level social capital, specifically community and institutional health, is significantly associated with higher mammography screening rates, particularly in non-metropolitan areas. These findings suggest that enhancing public trust and community engagement may improve screening behaviors. Future research should explore the role of social capital at multiple levels and its influence on various cancer screening behaviors.

## Highlights

• We examined associations between county-level social capital and mammography rates.
• Community and Institutional Health indices were associated with high rates.
• Associations with mammography rates were strongest in nonmetropolitan counties.

## 1 Introduction

Breast cancer is the most commonly diagnosed non-skin cancer among American women, accounting for approximately one in three newly diagnosed cancer cases (Siegel et al., 2023). The burden of breast cancer disproportionately affects older adults in the United States (U.S.), with the majority of cases diagnosed in women aged 50 and above (Giaquinto et al., 2022). Given the aging population in the U.S., identifying specific needs and effective strategies for prevention and early detection is crucial to reducing the burden of breast cancer among older adults. Mammography, the primary tool for breast cancer screening, plays a key role in breast cancer prevention and control in the U.S.

Social capital is broadly defined as a collective asset composed of social elements such as beliefs, trust, social networks, and civic engagement, which can enhance mutual support and cooperation among individuals and communities (Bhandari and Yasunobu, 2009). Numerous studies have demonstrated that social capital is associated with various health outcomes, such as reduced heart disease mortality and improved HIV treatment outcomes, as well as a positive relationship with beneficial health behaviors, such as vaccination for H1N1 (Lochner et al., 2003; Mukoswa et al., 2017; Jung et al., 2013). In relation to mammography, previous studies have found that individual-level social capital is positively associated with higher mammography rates (Shelton et al., 2016).

However, existing studies on social capital and mammography screening have largely focused on specific regional or racial/ethnic populations. For instance, a study conducted in Philadelphia, U.S. observed positive associations between perceived neighborhood-level social capital and mammography use (Dean et al., 2014). Additionally, many of these studies have relied on subjective measures of social capital in their analyses (Leader and Michael, 2013; Moudatsou et al., 2014). To our knowledge, no nationwide study has examined the relationship between social capital and mammography use specifically among older women. Therefore, the aim of this study is to investigate the relationship between social capital and mammography screening rates among older U.S. women using a standardized, nationwide, county-level social capital metric. We hypothesize that higher levels of county-level social capital will be positively associated with mammography rates.

## 2 Methods

This cross-sectional ecological study utilized deidentified and publicly available data and was therefore deemed exempt from review by the Emory University Institutional Review Board. The Strengthening the Reporting of Observational Studies in Epidemiology (STROBE) reporting guideline was followed (Vandenbroucke et al., 2014).

### 2.1 Data sources

#### 2.1.1 Screening mammography

County-level screening mammography data from 2018 were obtained from the Dartmouth Atlas of Health Care (DAHC), a dataset that analyzes and documents variations in how medical resources are distributed and used across the United States (Remington et al., 2015; University of Wisconsin Population Health Institute, 2021). DAHC utilizes data from the Centers for Medicare and Medicaid Services, the U.S. Census, the American Hospital Association, the American Medical Association, and the National Center for Health Statistics to provide contextual information on healthcare utilization. The county-level screening mammography measure captures the percentage of Medicare enrollees 67–69 years old per county who had at least one mammogram in 2018. The primary outcome of the study was a “high” mammography rate county, defined as a county that has a mammography rate that was in the top 10 % nationally. This designation was determined by assessing the 90th percentile cutoff point for county-level screening mammography rates.

#### 2.1.2 Social capital

Social capital indices were obtained from the Social Capital Project (Joint Economic Committee, 2018). Five social capital indices were included in the analyses: Family Unity, Institutional Health, Community Health, Collective Efficacy, and a composite Overall County-Level Social Capital index (Joint Economic Committee, 2018). The Family Unity subindex is comprised of indicators for family structure and cohesiveness (the share of births in the past year to unmarried women, the share of women ages 35–44 who are currently married and not separated, and the share of children living in a single-parent family, based on American Community Survey estimates). The Community Health subindex measures how robust community engagement is and consists of indicators measuring the share of adults who have engaged in volunteering, public meetings, and other community engagements, as well as the amount of membership and religious organizations. The Institutional Health subindex encompasses public trust in institutions and includes voting behavior indicators and the share of people who have confidence that corporations, the media, and public schools do what is right. The Collective Efficacy subindex comprises only one value, the violent crime rate - the lower the crime rate, the higher the collective efficacy because communities who have pro-social norms may do a better job of internal policing and community members may be less likely to violently harm other members. The Overall County-Level Social Capital index is a composite of the four subindices. Scores for each subindex were standardized and weighted using principal components analysis, and the overall county-level index was the weighted sum of the standardized subindex scores. All indices were determined at the county level.

Social capital indices were validated against the commonly used Rupasingha, Goetz, and Freshwater's county-level social capital measure, also called the Penn State Social Capital Index (Rupasingha and Goetz, 2008), and demonstrated strong correlations. Additionally, the Joint Economic Committee subindices were internally consistent with the overall index and demonstrated strong correlations with 50 county-level demographics, economics, education, and health indicators. While both the Penn State and the Joint Economic Committee indexes combine various forms of social capital, we selected the more-comprehensive Joint Economic Committee social capital measure because its data were more recently updated (2018 versus 2014).

We categorized the social capital indices into four groups based on quartile values. The fourth group (Q4) represented counties with values above the 75th percentile, the third group (Q3) included counties with values between the median and the 75th percentile, the second group (Q2) comprised counties with values between the 25th percentile and the median, and the first group (Q1) represented counties with values below the 25th percentile.

#### 2.1.3 Sociodemographic and healthcare covariates

County-level covariates were selected using directed acyclic graphs informed by prior literature. We included sociodemographic and clinical covariates known to be associated with screening mammography outcomes (Heller et al., 2018; Rosenkrantz et al., 2018). Sociodemographic and healthcare data from 2018 were obtained from the County Health Rankings and Roadmaps Program (CHR&R) (Remington et al., 2015; University of Wisconsin Population Health Institute, 2021). The CHR&R program provides data, tools, and guidance to improve community health and address health inequities. It annually ranks U.S. counties based on various health factors, such as social determinants, clinical care, and physical environment. The CHR&R program relies on a wide range of national and state-level data sources to measure health outcomes, including the Behavioral Risk Factor Surveillance System, U.S. Census Bureau, the National Center for Health Statistics, and the Centers for Medicare & Medicaid Services. Demographic covariates included age, racial/ethnic composition (percentages of Hispanic, Non-Hispanic Black, and Non-Hispanic White residents), median household income, and unemployment rates for people aged 16 and older. Healthcare access covariates included the rate of preventable hospital stays (hospital stays for ambulatory-care sensitive conditions per 100,000 Medicare enrollees) and the ratio of primary care physicians to the county population.

### 2.2 Statistical analysis

Descriptive statistics, including means and standard deviations, were reported for all variables. Continuous variables were compared using *t*-tests and analysis of variance.

Multivariable log-binomial regression analysis was used to estimate adjusted Prevalence Ratios (aPRs) and 95 % confidence intervals for “High” mammography rates, after adjusting for county-level sociodemographic and healthcare covariates. For each subindex, aPRs and CIs were calculated by comparing the proportion of high mammography rate counties in the top quartile (Q4) with the lowest quartile (Q1) as the reference group. Additionally, we conducted stratified analyses to explore the associations between social capital and screening mammography by county metropolitan status. The 2013 Rural–Urban continuum codes (RUCC) were used classify counties as metropolitan (RUCC 1–3) or nonmetropolitan (RUCC 4–9) based on population and degree of urbanization (U.S. Department of Agriculture, 2013). A total of 2765 counties were included in the final analytical dataset, after removing counties with missing social capital (*n* = 93) and covariate (*n* = 284) data from the original sample of 3142 counties. All statistical analyses were performed using R software, version 4.3.2 (Vienna, Austria).

## 3 Results

### 3.1 Descriptive characteristics

The descriptive characteristics of U.S. counties, stratified by metropolitan status, are shown in Table 1. County-level mammography screening rates ranged from 17 % to 64 %, with a mean of 41 % (standard deviation = 7.5 %). Metropolitan counties exhibited a higher screening mammography rate (43 %) compared to non-metropolitan counties (40 %). Compared with non-metropolitan counties, metropolitan counties had a lower proportion of residents aged 65 years and older and a higher percentage of Non-Hispanic Black and Hispanic residents. Additionally, metropolitan counties reported a higher median household income and slightly lower unemployment rates than non-metropolitan counties. Healthcare access and utilization metrics further highlighted disparities between metropolitan and non-metropolitan counties. Metropolitan counties had fewer preventable hospital stays per 100,000 Medicare enrollees compared to non-metropolitan counties and the ratio of primary care physicians to the county population was also higher in metropolitan counties than in non-metropolitan counties. All comparisons between metro and non-metro counties' means for covariates and mammography rates were significantly different (all *p* < 0.001).

**Table 1:** Descriptive Characteristics of United States Counties by Metropolitan Status (2018).

Characteristic, mean (SD)	All Counties *n* = 2765 counties	Metropolitan Counties^b^ *n* = 1107 counties	Nonmetropolitan Counties^b^ *n* = 1658 counties	*p* value^c^
Mammography				
Screening mammography rate^a^ (%)	41.18 (7.47)	42.70 (6.05)	40.16 (8.13)	<0.001
Sociodemographic				
Age > 65 years (%)	19.03 (4.51)	17.12 (4.11)	20.30 (4.31)	<0.001
Race/Ethnicity				<0.001
Non-Hispanic White (%)	76.35 (19.41)	72.96 (18.85)	78.61 (19.46)	
Non-Hispanic Black (%)	9.07 (13.99)	11.04 (13.24)	7.76 (14.32)	
Hispanic (%)	9.68 (13.55)	10.48 (12.89)	9.15 (13.95)	
Annual Median Household Income	$53,390 ($14,040)	$61,136 ($16,010)	$48,217 ($9532)	<0.001
Unemployment (%)	4.09 (1.37)	3.92 (1.19)	4.20 (1.46)	<0.001
Healthcare				
Rate of Preventable Hospital Stays per 100,000 Medicare enrollees	4600 (1685)	4483 (1296)	4678 (1897)	<0.001
Ratio of Primary Care Physicians to the County Population (per 10,000 people)	5.50 (3.42)	6.27 (3.92)	4.98 (2.94)	0.001
Social Capital Index				
Overall Social Capital^d^	‐0.01 (0.99)	−0.16 (0.92)	0.09 (1.02)	<0.001
Community Health^e^	0.11 (0.87)	−0.54 (0.61)	0.17 (0.90)	<0.001
Family Unity^f^	0.02 (0.97)	0.07 (0.89)	−0.01 (1.02)	<0.001
Institutional Health^g^	0.00 (0.99)	0.15 (0.93)	−0.1 (1.02)	<0.001
Collective Efficacy^h^	−0.03 (1.00)	−0.24 (1.08)	0.11 (0.91)	<0.001

a Percentage of enrollees with at least one screening mammogram in 2018.

b Counties were classified according to the 2013 U.S. Department of Agriculture Rural–Urban continuum Codes (RUCC) as metropolitan (RUCC 1–3) or nonmetropolitan (RUCC 4–9).

c P value for ANOVA or chi-square test.

d Composite measure that assesses social capital within a county based on four subindices: Family Unity, Community Health, Institutional Health, and Collective Efficacy.

e Subindex that measures community interactions and civic engagement within a county.

f Subindex that measures the strength and stability of family structures within a county.

g Subindex that measures the strength and trustworthiness of local institutions and organizations within a county.

h Subindex that measures a community's ability to maintain social order and safety within a county.

Social capital indicators also differed significantly by metropolitan status. While metropolitan counties scored lower on Overall Social Capital, Community Health, and Collective Efficacy, they exhibited slightly higher Family Unity and Institutional Health scores compared to non-metropolitan counties (all *p* < 0.001).

### 3.2 Associations between Social Capital and Screening Mammography Rates

Table 2 presents the prevalence ratios and 95 % confidence intervals for “high” mammography rates across social capital quartiles, stratified by all counties, metropolitan counties, and nonmetropolitan counties. For all counties, higher social capital was significantly associated with increased mammography rates. Specifically, counties in the highest quartile (Q4) of Overall Social Capital had an aPR of 2.29 (95 % CI: 1.20–4.36) for high mammography rates compared to the lowest quartile (Q1). Counties in the highest quartile of Community Health had an aPR of 1.99 (95 % CI: 1.25–3.17) and those in the highest quartile of Institutional Health had an aPR of 4.31 (95 % CI: 2.40–7.75) when compared to their respective lowest quartiles.

**Table 2:** Prevalence Ratios and 95 % Confidence Intervals for “High” Screening Mammography Rate in All, Metropolitan, and Nonmetropolitan United States Counties (2018).

Social Capital Index	All Counties aPR^b^ (95 % CI)	Metropolitan Counties^a^ aPR^b^ (95 % CI)	Nonmetropolitan Counties^a^ aPR^b^ (95 % CI)
Overall Social Capital^c^			
Q4	2.29 (1.20–4.36)	2.08 (0.69–6.32)	3.57 (1.46–8.71)
Q3	1.28 (0.69–2.38)	1.79 (0.64–5.03)	1.65 (0.70–3.89)
Q2	1.50 (0.83–2.73)	1.97 (0.71–5.44)	1.53 (0.67–3.50)
Q1	1.0	1.0	1.0

Community Health^d^			
Q4	1.99 (1.25–3.17)	1.96 (0.93–4.13)	3.19 (1.50–6.78)
Q3	1.80 (1.15–2.82)	1.42 (0.66–3.07)	2.71 (1.33–5.55)
Q2	1.13 (0.70–1.85)	1.94 (0.89–4.24)	1.59 (0.75–3.36)
Q1	1.0	1.0	1.0

Family Unity^e^			
Q4	0.90 (0.56–1.43)	0.57 (0.26–1.26)	1.23 (0.67–2.24)
Q3	1.01 (0.65–1.59)	0.64 (0.31–1.31)	1.34 (0.74–2.44)
Q2	0.96 (0.61–1.51)	0.99 (0.51–1.93)	0.93 (0.50–1.74)
Q1	1.0	1.0	1.0

Institutional Health^f^			
Q4	4.31 (2.40–7.75)	2.70 (1.11–6.60)	9.25 (3.57–23.97)
Q3	1.47 (0.81–2.68)	1.35 (0.55–3.31)	2.72 (1.03–7.14)
Q2	1.50 (0.82–2.77)	1.55 (0.64–3.80)	2.27 (0.84–6.15)
Q1	1.0	1.0	1.0

Collective Efficacy^g^			
Q4	1.18 (0.75–1.85)	0.70 (0.34–1.45)	1.76 (1.00–3.10)
Q3	1.10 (0.71–1.72)	0.76 (0.38–1.55)	1.31 (0.74–2.33)
Q2	1.27 (0.82–1.99)	0.92 (0.47–1.83)	1.38 (0.77–2.46)
Q1	1.0	1.0	1.0

Note: “High” screening mammography rate = mammography rate that was greater than the top 10 % nationally.

a Counties were classified according to the 2013 U.S. Department of Agriculture Rural–Urban Continuum Code (RUCC) classification scheme as metropolitan (RUCC 1–3) or nonmetropolitan (RUCC 4–9).

b Prevalence Ratios adjusted for age, race/ethnicity, annual median household income, unemployment, rate of preventable hospital stays, and the ratio of primary care physicians to the county population.

c Composite measure that assesses social capital within a county based on four subindices: Family Unity, Community Health, Institutional Health, and Collective Efficacy.

d Subindex that measures community interactions and civic engagement within a county.

e Subindex that measures the strength and stability of family structures within a county.

f Subindex that measures the strength and trustworthiness of local institutions and organizations within a county.

g Subindex that measures a community's ability to maintain social order and safety within a county.

When examining metropolitan and nonmetropolitan counties separately, the results reveal distinct patterns. In nonmetropolitan counties, the associations between social capital and mammography rates exhibited stronger and more consistent associations. For instance, nonmetropolitan counties in the highest quartiles of Overall Social Capital (aPR = 3.57,95 % CI: 1.46–8.71), Community Health (aPR = 3.19, 95 % CI: 1.50–6.78), and Institutional Health (aPR of 9.25, 95 % CI: 3.57–23.97) demonstrated strong associations with “high” mammography rates when compared to their respective lowest quartiles (Table 2).

## 4 Discussion

This study investigated the associations between county-level social capital and screening mammography rates among older women in the United States. Our results highlight the importance of examining social capital subindices. We found that the highest quartile of “overall” county-level social capital was associated with higher screening mammography rates. Additionally, when examining social capital subindices, we observed significant associations between screening mammography rates and the social capital indices of Community Health and Institutional Health. These associations were strongest in nonmetropolitan counties.

The potential mechanisms linking county-level social capital to screening mammography rates are multifaceted and likely involve a combination of social networks, community norms, and trust in healthcare systems. For example, we observed significant associations between the Community Health subindex and screening mammography rates. This subindex included indicators of community engagement, such as participation in volunteering activities, civic engagement, and involvement with religious organizations. Our results are consistent with similar studies that have examined the relationships between civic engagement and breast and cervical cancer screening, as well as religious service attendance and cancer screening (Moudatsou et al., 2014; Hill et al., 2006; Salmoirago-Blotcher et al., 2011). However, while there is qualitative evidence supporting a relationship between volunteerism and cancer screening, prior quantitative data on volunteering and cancer screening are sparse (Molina et al., 2018). A potential explanation for our Community Health findings is that involvement in community and religious activities may enhance health awareness, increase access to resources, and provide social support for health-promoting behaviors, such as preventive healthcare. Similarly, individuals who engage in volunteering may be more likely to participate in cancer screening, as volunteering can foster a sense of social responsibility, increased health awareness, and greater access to health information and resources through community networks.

We also observed associations between the Institutional Health subindex and screening mammography rates. The Institutional Health subindex serves as a proxy for public trust in institutions (e.g., voting behavior and confidence that corporations, the media, and public schools act ethically). To our knowledge, few U.S. studies have examined the relationship between political participation and screening mammography. However, previous studies in Europe and Australia have reported links between voting behavior and cancer screening. For instance, a population-based survey of adult women in England found that women who did not attend cervical cancer screenings were more likely to be disillusioned with public services, as indicated by a lower likelihood of voting in elections. This association remained significant even after controlling for common barriers to screening, such as embarrassment and fear of pain (Waller et al., 2009). A follow-up study using computer-assisted interviews found that women who were not registered to vote were more likely to be overdue for cervical cancer screening (Macedo et al., 2012). Similarly, a qualitative study conducted in Australia found through participant interviews that institutional mistrust (e.g., mistrust in the government, medical system, and local health services) played a large role in individuals' decisions to forgo participation in the national colorectal cancer screening program (Ward et al., 2015). Additionally, an online survey conducted in the Netherlands showed that trust in government and national screening programs significantly influenced positive attitudes toward colorectal cancer screening (Douma et al., 2018). While the exact mechanisms between institutional trust and cancer screening are still unclear, it may be that individuals who lack public trust in institutions would be less likely to trust medical advice and adhere to recommended screening guidelines set by agencies (e.g., U.S. Preventive Services Task Force and American Cancer Society) that receive support from governmental institutions (Lasser et al., 2008). These findings, along with our current analysis, suggest that understanding the relationship between public trust in institutions and screening behaviors could inform community-level screening programs and interventions.

It should be noted that our study revealed stronger associations in non-metropolitan U.S. counties compared to metropolitan counties. We hypothesize that this is because non-metropolitan communities may have closer-knit social networks, higher levels of trust, and stronger interpersonal connections, while metropolitan areas, though more diverse and resource-rich, may experience weaker social ties due to larger populations and greater mobility. Our study was unable to evaluate the intensity of these social capital subindices in U.S. counties. Therefore, future longitudinal studies are needed to better understand potential urban-rural differences in social capital intensity and mechanisms between social capital dimensions and preventive screening behaviors.

Strengths of this study include its nationwide scope, with a large sample of 2765 U.S. counties, and the use of Medicare data for mammography screening rates, providing standardized and accurate outcome measurements. However, this study has several limitations. As a cross-sectional ecological study, it cannot establish causality between social capital and mammography rates. Screening mammography rate data reflect only one year, 2018. Additionally, the reliance on secondary data sources also poses challenges, as these data may not fully capture the potential regional differences and nuances of social capital or other relevant factors. Although this study focused on older women on Medicare, future research could replicate or expand these analyses to examine whether similar patterns hold for younger women or women not enrolled in Medicare. Furthermore, some county-level covariate data were missing, which reduced the analytical sample size. Lastly, unmeasured confounders, such as past cancer screening behaviors, were not available in the study data sources.

In conclusion, we observed significant associations between county-level community and institutional health and screening mammography among older women in the United States. Future research should explore social capital domains at the individual, neighborhood, and county levels to understand their impact on mammography screening, particularly in non-metropolitan U.S. areas. While this study specifically focused on mammography rates, future studies are warranted to examine social capital's role across various screening behaviors for different cancers.

## Funding

This work was supported, in part, by the National Cancer Institute under award number P30CA138292 and the National Institute of Environmental Health Sciences under Award Number P30ES019776.

## CRediT authorship contribution statement

**Tracy Huang:** Writing – review & editing, Writing – original draft, Visualization, Software, Formal analysis, Data curation. **Christine C. Ekenga:** Writing – review & editing, Writing – original draft, Supervision, Funding acquisition, Conceptualization.

## Declaration of competing interest

The authors declare that they have no known competing financial interests or personal relationships that could have appeared to influence the work reported in this paper.

## Data Availability

Data will be made available on request.
